# Hanging bladder stone due to misplaced surgical suture several years after hysterectomy: A case report

**DOI:** 10.1016/j.ijscr.2021.106586

**Published:** 2021-11-15

**Authors:** Andrey Satria Julian, Ahmad Agil

**Affiliations:** Division of Urology, Faculty of Medicine University of Padjadjaran, Hasan Sadikin Hospital Bandung, Indonesia

**Keywords:** Bladder stone, Suture, Iatrogenic

## Abstract

**Introduction and importance:**

Urinary bladder calculi are rarely seen in women and any history of previous pelvic surgery must, therefore, raise suspicion of an iatrogenic etiology. The incidence of iatrogenic foreign bodies rise recently due to increasing number of surgical procedure. According to the literature, fewer than 2% of all bladder calculi occur in female subjects and, thus, their presence should provoke careful assessment of the etiology. There have been a number of reports on bladder calculi in women with a history of gynecologic procedures. We report a case of bladder calculus after hysterectomy that was treated successfully by open surgery_._

**Case presentation:**

A 54 year old lady with dysuria, had a history of urinary stone for one last year. She was complaining hematuria. She had radical hysterectomy 20 years ago and cystoscopy Lithotripsy because of bladder stone a year ago. Physical examination was unremarkable. Abdominal CT scan with contrast had revealed multiple vesicolithiasis and irregular calcifications attached to the superior aspect of vesical urinary.

**Clinical discussion:**

Cystoscopic evaluation was performed and confirmed presence of calculi forming around several surgical sutures fixed to the bladder wall. The intravesical calculus had developed from non-absorbable sutures and hung on the dome of the urinary bladder. The stone and residuum of the suture were retrieved by performing an open surgery.

**Conclusion:**

The presence of an intravesical stone should be suspected in patients with a history of hysterectomy who have symptoms in the lower urinary tract. A hanging stone on the dome of the urinary bladder implies that non absorbable suture materials intrusion into the urinary bladder. The complication can be prevented by the routine use of absorbable material and doublechecking with cystoscopy.

## Highlights

• The incidence of iatrogenic foreign bodies rise recently due to increasing number of surgical procedure.
• According to the literature, fewer than 2% of all bladder calculi occur in female subjects
• The presence of an intravesical stone should be suspected in patients with a history of hysterectomy
• A hanging stone on the dome of the urinary bladder implies that non absorbable suture materials intrusion in bladder
• There have been a number of reports on bladder calculi in women with a history of gynecologic procedures

## 1 Introduction

According to the literature, bladder stones rarely occur in females. Hence the occurrence of bladder stones in careful assessment of the disease etiology [1]. Most bladder stones occur as a result of bladder outlet obstruction and, rarely, foreign body intrusion which causes urinary stasis. In the case of female patients, bladder obstruction could happen secondary to previous gynecologic conditions resulting in urethral kinking [2]. In addition, several investigators have reported that intrauterine contraceptive devices migrate through the wall of the urinary bladder and then serve as a nidus of intra vesical stone formation [3], [4], [5], [6]. Hence, the occurrence of bladder calculi in patients with history of such procedures, should raise a suspicion of iatrogenic etiology.

Identification of bladder stone is usually done through serial kidney-ureter-urinary bladder (KUB) X-ray films for radiopaque stones and ultrasonography for radiolucent stones. Bladder stones usually move when the patient is repositioned. In some cases, however, bladder stones are immovable. Most of these stones are found during reconstructive procedures for urinary bladder [7]. However, immovable bladder stone is quite rare in patients who have never had reconstructive surgery of the urinary bladder. This article reports the case of a woman who developed an immovable intra vesical stone 20 years after hysterectomy.

## 2 Methods

This study is based on a case from our urology clinic which is inspected with relevant literatures. Consent from the patient has been obtained for this study. This case has been reported in accordance to the SCARE Guideline [8].

## 3 Case presentation

A 54 years old woman came to our urology clinic with a chief complaint of dysuria for more than a year prior to admission. She also complained hematuria, urgency and increase of urinary frequency. The patient had a history of radical hysterectomy for myoma uterine 20 years earlier with benign pathological result. Radiotheraphy and chemotheraphy was denied.

**Fig. 1:**
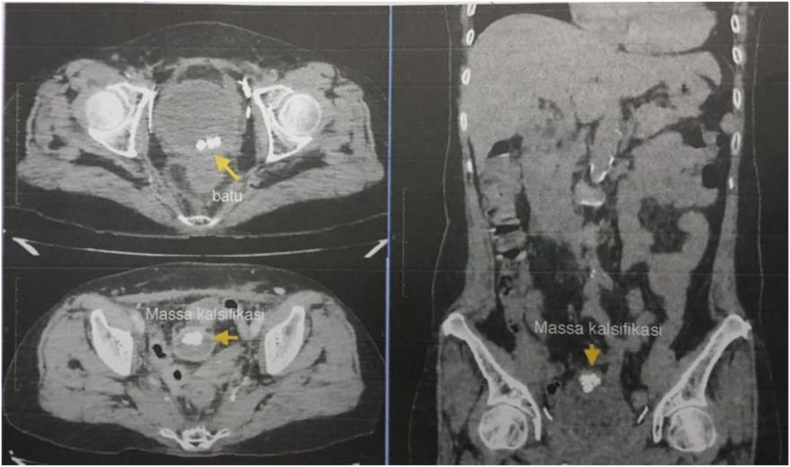
Non-contrast abdominal pelvic CT Scan.

There were history of spontaneous stone passage, sandy urine, and cloudy urine. The patient was fully conscious with stable hemodynamic status, conjunctiva were not anemic and sclera no jaundice. Laboratory studies revealed Hb of 10.9 g/dl, white blood counts of 11.560/ul, blood urea nitrogen of 21.0 g/dl, blood creatinine of 0.6 mg/dl. Urinalysis showed >50 white blood cells per high power field, and >50 blood cells per high power field. Non-contrast abdominal pelvic computed tomography was conducted with confirmed multiple vesicolithiasis (±6, 7.7 and 9.4 mm in diameter, 790–920 HU) and irregular calcifications attached to the superior aspect of vesical urinary size ±11 × 18 × 15 mm, 677–855 HU (Fig. 1).

**Fig. 2:**
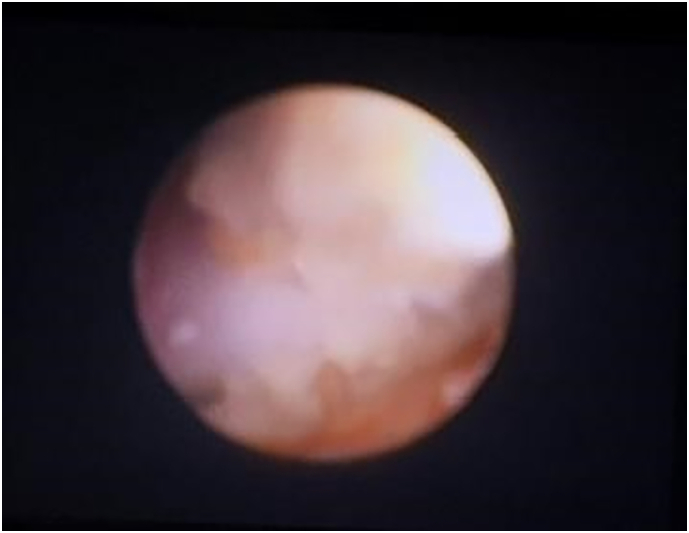
Cystoscopy, Cahya Kawaluyan Hospital, 18-20-2019.

**Fig. 3:**
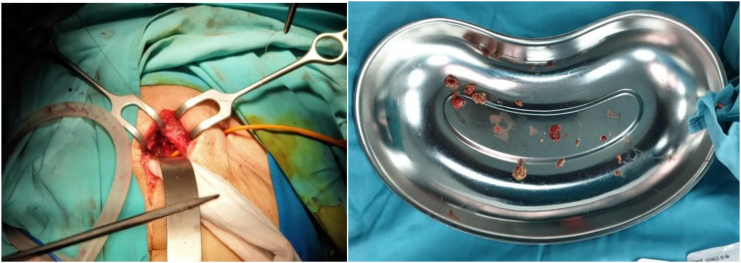
The stone and residuum of the suture were retrieved by performing an open surgery (Left) and Stone fractures and suture material after procedure (right).

Cystoscopy showed a hanging stone on the dome of the urinary bladder (Fig. 2). Our first attempt using pneumatic lithoclast ended in failure because the sutures could not be removed. Considering the risk of bladder dome perforation, open surgery for removal of the sutures and the residual stones was performed (Fig. 3).

## 4 Discussion

Bladder calculi account for 5% of all urinary calculi and usually occur due to bladder neck outlet obstruction, indwelling foreign bodies, or urinary tract infection. Bladder calculi occur more frequently in males, across nations, races, and age groups. Females account for only 2% of all patients. The frequency of occurrence increases yearly after the age of 50 years [1]. Men with prostate disease or have had previous prostate surgery and women who have undergone anti-incontinence surgery are at higher risk for developing bladder calculi. Patients with spinal cord injuries are also at higher risk for developing bladder calculi [2].

Bladder calculi are uncommon in women and any history of previous pelvic surgery must, therefore, raise the suspicion of an iatrogenic origin [9]. The etiology of foreign bodies in the bladder may be classified as follows: insertion of a foreign body by the person himself or herself (for the purpose of masturbation, curiosity, or psychiatric or senile reasons), iatrogenic (such as penetrating injury during surgery of the urinary tract, uterus, vagina, or rectum), and migration of an intrauterine device. These foreign bodies act as a nidus for stone formation in the bladder. Some reports in the literature showed that bladder stone formation was the result of complication following surgeries [10].

Endoscopy is an effective and safe method for removing bladder stones. However, a bladder stone resulting from a foreign body that has become fixed on the bladder wall may require an open surgery for its removal [11]. The main principle for treating a bladder stone is to remove the underlying cause of stone formation, such as bladder outlet obstruction or bladder infection. According to the literature, bladder stones caused by foreign bodies involving sutures contain components such as calcium, ammonium, phosphate, oxalate, and uric acid. These components mostly exist as mixtures [11], [12].

A hanging intravesical stone on the dome of urinary bladder is rare and usually associated with procedures on the organs surrounding the urinary bladder, for example, hysterectomy or the placement of intrauterine devices [13], [14]. Relatively speaking, the incidence of sutures which served as a nidus of stone formation is lower than that of intrauterine devices. In both cases, the investigators found that synthetic and non-absorbable suture materials were encrusted and become a bladder stone. The underlying mechanism would be the suture penetrate through the dome of urinary bladder and then caused the deposition of calcium salts [15].

Abdominal radiography may reveal most intravesical stones. However, radiolucent stones would be missed by a plain X-ray film. Ultrasonography is useful for primary evaluation of intravesical stone formation [16]. In our patient, ultrasonography revealed that the bladder stone hangs from the dome of the urinary bladder instead of accumulating at the bottom. This implied the existence of a foreign body which acts as a nidus. We suspect that, in our patient, the non-absorbable suture had been inadvertently placed transvesically during hysterectomy, and was not detected intraoperatively. It is well recognized that non-absorbable suture material can act as a nidus for stone formation following perivesical surgery. This serves a reminder that the evaluation of recurrent cystitis with bladder calculi in women demands careful attention to the history of previous pelvic surgery.

## 5 Conclusion

A hanging intravesical stone on the dome of the urinary bladder is rare. It usually hints that the bladder stone was derived from encrustation of suture material. The complication can be prevented by the routine use of absorbable material in sutures outside the urinary bladder, no use of any suture through the urinary bladder and double-checking with cystoscopy.

## Sources of funding

None declared.

## Ethical approval

None declared.

## Research registration

N/a.

## Provenance and peer review

Not commissioned, externally peer-reviewed.

## Guarantor

dr. Ahmad Agil, Sp.U(K)

## Consent

Written informed consent was obtained from the patient for publication of this case report and accompanying images. A copy of the written consent is available for review by the Editor-in-Chief of this journal on request

## CRediT authorship contribution statement

**Andrey Satria Julian:** Conceptualization, Methodology, Software, Validation, Formal analysis, Investigation, Resources, Data curation, Writing – original draft, Writing – review & editing, Visualization, Supervision. **Ahmad Agil:** Conceptualization, Methodology, Software, Validation, Formal analysis, Investigation, Resources, Data curation, Writing – original draft, Writing – review & editing, Visualization, Supervision.

## Declaration of competing interest

None declared.

## References

[1] Drach G.W. (1992).

[2] Schwartz B.F., Stoller M.L. (2000). The vesical calculus. Urol. Clin. North Am..

[3] Atakan, Rfan H., Kaplan M., Ertrk E. (2002). Intravesical migration of intrauterine device resulting in stone formation. Urology.

[4] Dede F.S., Dilbaz B., Sahin D., Dilbaz S. (2006). Vesical calculus formation around a migrated copper-T 380-a. Eur. J. Contracept. Reprod. Heal. Care.

[5] Demirci D., Ekmekçioglu O., Demirtaş A., Gülmez I. (2003). Big bladder stones around an intravesical migrated intrauterine device. Int. Urol. Nephrol..

[6] Khan Z.A., Khan S.A., Williams A., Mobb G.E. (2006). Intravesical migration of levonogestrel-releasing intrauterine system (LNG-IUS) with calculus formation. Eur. J. Contracept. Reprod. Heal. Care.

[7] Cursio R., Choquenet C. (2002). Iatrogenic bladder stone formation on absorbable suture 3-years after radical prostatectomy. Minerva Urol. Nefrol..

[8] Agha R.A. (2020). The SCARE 2020 guideline: updating consensus Surgical CAse REport (SCARE) guidelines. Int. J. Surg..

[9] Evans J.W.H., Chapple C.R., Ralph D.J., Milroy E.J.G. (1990). Bladder calculus formation as a complication of the Stamey procedure. Br. J. Urol..

[10] Yalçin V., Demirkesen O., Alici B., Önol B., Solok V. (1998). An unusual presentation of a foreign body in the urinary bladder: a migrant intrauterine device. Urol. Int..

[11] Lipke M., Schulsinger D., Sheynkin Y., Frischer Z., Waltzer W. (2004). Endoscopic treatment of bladder calculi in post-renal transplant patients: a 10-year experience. J. Endourol..

[12] Su S.-T., Huang H.-F., Chang S.-F. (2009). Encrusted bladder stone on non-absorbable sutures after a cesarean section: a case report. JTUA.

[13] Özgür A. (2004). Intravesical stone formation on intrauterine contraceptive device. Int. Urol. Nephrol..

[14] Litschgi M., Benz J., Glatthaar E. (1975). Bladder stones as a complication of gynecologic surgery in German. Frotschr. Med..

[15] Shah I., Gupta R., Gupta C.L. (2006). Hanging calculi in urinary bladder- retrival by endoscopic means. JK Pract..

[16] Mahmutyazcolu K., Özdemir H., Özkan P. (2002). Migration of an intrauterine contraceptive device to the urinary bladder: sonographic findings. J. Clin. Ultrasound.

